# A Topology-Aware Extension of Guttae Area Ratio in Fuchs Endothelial Corneal Dystrophy

**DOI:** 10.1167/tvst.15.8.11

**Published:** 2026-08-18

**Authors:** Molly Orlick, Henry Bair

**Affiliations:** 1 Sidney Kimmel Medical College, Philadelphia, PA, USA; 2 Wills Eye Hospital, Philadelphia, PA, USA e-mail: henry.c.bair@gmail.com

We read with interest the study by Sarin and colleagues,^1^ which validates automated measurement of guttae area ratio (GAR) in Fuchs endothelial corneal dystrophy (FECD). By showing equivalence between automated and expert manual GAR and demonstrating increasing GAR across modified Krachmer grade (mKg), the authors provide an important tool for objective FECD phenotyping. Building on prior quantitative endothelial imaging work,^2^^–^^4^ this is timely as pre-edema trial endpoints become increasingly relevant. Because GAR is computed from a guttae segmentation mask, the platform also creates an opportunity to study guttae architecture after the segmentation has been validated as a measure of area fraction.

The mKg 5 subanalysis illustrates this opportunity. In mKg 4 images, definite subclinical edema by Scheimpflug tomography was associated with higher GAR; in mKg 5 images, mean GAR was nearly identical without versus with definite subclinical edema (62.02% vs. 61.30%; *P* = 0.86).^1^^,^^5^ This pattern is compatible with a ceiling in the discriminative value of area fraction in advanced FECD. Once guttae occupy approximately 60% of the analyzed specular field, small increments in total guttae area may become less informative about tomographic edema status and may serve as an imperfect proxy for residual endothelial functional reserve.

Although Sarin and colleagues^1^ appropriately note that guttae size and confluence may matter, their validated segmentation mask makes this observation measurable. In advanced FECD, disease may shift from scattered guttae to connected guttae plaques that divide the visible endothelial mosaic into smaller guttae-free regions. Two eyes with identical GAR could therefore differ clinically: one may contain dispersed, nonconfluent guttae with large contiguous guttae-free areas, whereas another may contain a confluent plaque that fragments the remaining mosaic into small, isolated islands. This topology-aware view is biologically plausible because the guttae–cell interface is a site where endothelial stress, migration, and survival may be particularly relevant.^6^

These architectural features could be derived from the same segmentation masks without new imaging hardware. Intuitive topology classes include connectedness of the guttae mask, quantified by the largest connected component or spanning-cluster status; fragmentation of visible guttae-free regions, quantified by island count and island-size distribution; proximity of guttae-free regions to guttae, quantified by distance transforms; and interface burden, quantified by boundary length or edge density. These measures should be evaluated alongside GAR, not as replacements for it, and any composite index should be developed only after individual features demonstrate incremental validity.

The authors’ central–peripheral analysis also argues for spatial standardization. Central fixation images had substantially higher GAR than peripheral fixation images, with a mean difference of 33.88 percentage points, exceeding reported grade-to-grade differences in central GAR.^1^ Thus, GAR is best interpreted as a location-specific image-field metric unless acquisitions are anatomically standardized, registered, montaged, or explicitly labeled by fixation location. In multicenter natural history or therapeutic studies, field placement could otherwise masquerade as progression or treatment response.

Longitudinal GAR also requires a measurement error framework. The reported intraclass correlation coefficient of 0.85 supports repeatability, but the Bland–Altman limits for repeat images were ±26.36 percentage points; using the reported within-session SD of 9.51, this corresponds closely to a 95% minimal detectable change of approximately 26.4 percentage points for two independent single-image measurements.^1^ The authors’ aligned longitudinal example, in which GAR increased by 6.06 percentage points over 71 days, is useful as proof of concept. For future natural history or therapeutic endpoints, however, ΔGAR should be accompanied by same-field, registration-specific repeatability, image-averaged estimates, or confidence intervals around change.

A practical next step would be an exploratory reanalysis of the existing mKg 4 to 5, edema-labeled image set using models that account for clustering by eye and participant. Such an analysis could compare GAR alone, topology alone, GAR plus topology, and GAR plus topology plus image location for prediction of definite subclinical edema, with attention to discrimination, calibration, and incremental value. We hypothesize that topology will add the greatest information in mKg 5 images, where scalar GAR appears to lose discriminatory power. Sarin and colleagues^1^ have provided a valuable quantitative foundation; the next opportunity is to move from guttae burden to guttae architecture, integrating area, topology, location, and detectable change into a more clinically informative computational endothelial phenotype.
